# Aging and Fracture Resistance of Implant-Supported Molar Crowns with a CAD/CAM Resin Composite Veneer Structure

**DOI:** 10.3390/jcm12185997

**Published:** 2023-09-15

**Authors:** Angelika Rauch, Wendy Heinzmann, Martin Rosentritt, Sebastian Hahnel, Michael Benno Schmidt, Florian Fuchs, Andreas Koenig

**Affiliations:** 1 Department of Dental Prosthetics, University Hospital Regensburg, Franz-Josef-Strauß-Allee 11, 93053 Regensburg, Germany; martin.rosentritt@ukr.de (M.R.); sebastian.hahnel@ukr.de (S.H.); michael3.schmidt@ukr.de (M.B.S.); 2 Zahnärzte im Leipziger Westen, Karl-Heine-Straße 26, 04229 Leipzig, Germany; wendyheinzmann@gmx.de; 3 Department of Prosthetic Dentistry and Dental Materials Science, Leipzig University, Liebigstr. 10-12, Haus 1, 04103 Leipzig, Germany; florian.fuchs@medizin.uni-leipzig.de (F.F.); andreas.koenig@medizin.uni-leipzig.de (A.K.)

**Keywords:** chipping, Cad-on, dental ceramic, PEEK, polymers, digital veneering

## Abstract

Chipping of implant-supported molar crowns (iSCs) is a frequently reported complication. This study aimed to investigate the in-vitro aging and fracture resistance of iSCs with a CAD/CAM resin composite veneer structure fabricated with the Rapid Layer Technology (RLT) approach. Eight iSCs per group were fabricated by using two different CAD/CAM resin composites (Shofu Block HC: SH; Grandio blocs: GB) for veneer structures, and zirconia (ZrO_2_), polyetheretherketone (PEEK), and cobalt–chromium (CoCr; control) as framework materials. The surfaces to be bonded were sandblasted, cleaned in an ultrasonic bath, and a coupling agent was applied. A self-adhesive resin luting composite was used to adhesively lute the veneer structures to the frameworks. The crowns were semi-permanently cemented to the abutments. After storage in deionized water, iSCs were loaded in a chewing simulator (TCML, 10,000 thermal cycles 5 °C to 55 °C for 20 s, 1.2 million, loading force 50 N). Four ZrO_2_ and one CoCr crown did not survive the TCML. The fracture force was determined after 24 h storage in deionized water and yielded values of ≥974 N. Lowest fracture forces were yielded in the PEEK-SH group in comparison to CoCr or ZrO_2_ groups (*p* ≤ 0.031). For identical framework materials, no significant influence of the veneering material was observed. All PEEK-GB frameworks fractured, and chipping occurred for ZrO_2_-SH and all CoCr frameworks. PEEK-SH and ZrO_2_-GB presented both chipping and framework fractures. Within the limitations of this in-vitro study, the RLT with a CAD/CAM resin composite veneer structure might be a promising approach to veneer iSCs. Yet, the choice of the CAD/CAM resin composite and of the framework material determine the fracture resistance.

## 1. Introduction

The veneering of implant-supported single crowns (iSC) is very popular in daily dental practice [1]. For porcelain-fused-to-metal or veneered zirconia iSCs, the estimated survival rates are clinically acceptable. Yet, ceramic chipping is a frequently reported complication, especially with zirconia frameworks, in which the location of the implant does not significantly impact the occurrence of chipping [2,3]. To avoid chipping, modifications of the veneering ceramic, e.g., its fabrication technique or even a monolithic approach might be applied. However, the application of monolithic zirconia for iSCs is critically discussed since an occlusal overload on the strong zirconia material might fatally damage the implant [4]. Thus, other tooth-colored materials have been investigated like polyaryletherketones (PAEK), especially polyetheretherketone (PEEK) that are featured by mechanical properties closer to the natural tooth. Compared to the natural tooth structure (E-modulus 9–25 GPa), PEEK is characterized by a more similar elasticity (elastic modulus 3–4 GP) than zirconia (210 GPa) [5,6]. PEEK materials for the fabrication of tooth-colored fixed partial dentures have esthetical shortcomings. Therefore, the veneering of PEEK is recommended [7] but a conventional veneering technique is prone to cracks [8].

A digital veneering that is milled from a tooth-colored material might be favorable [8]. The Rapid Layer Technology (RLT) implies the CAD/CAM fabrication of a milled veneer structure that is adhesively bonded to a framework. Some in-vitro studies have already investigated the aging and/or fracture behavior of veneer structures from silica-based materials [9,10,11,12]; however, information on other esthetical tooth-colored materials for veneer structures in an RLT approach is scarce [8,13].

**Figure 1:**
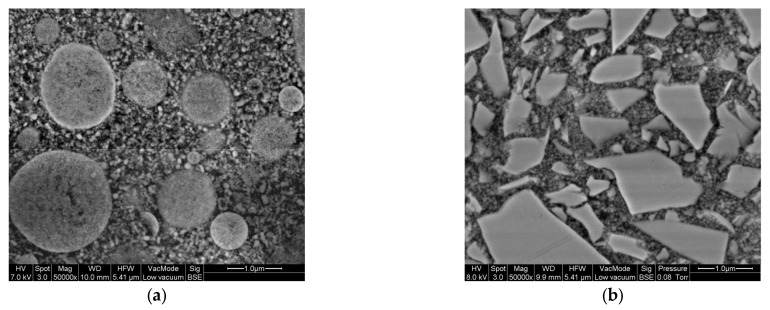
Scanning electron microscope images of the veneering materials (Quanta 400 FEG, FEI Company, Hillsboro, OR, USA) with magnifications of 50,000× at 7 kV and 8 kV in Back-Scattered Electron (BSE) mode under low vacuum (**a**) Shofu Block HC; (**b**) Grandio blocs.

For CAM-milled veneer structures, indirect resin composites (CAD/CAM resin composites) might be an interesting alternative to silica-based materials, since their elastic modulus with values between 8 to 18 GPa is closest to that of natural teeth [6], and CAD/CAM resin composites induce lower wear of antagonist teeth [14,15]. However, CAD/CAM resin composites are very heterogeneous in terms of composition, filler content, and filler content size, which might determine the mechanical properties such as the elastic modulus or Vickers hardness. Therefore, when using CAD/CAM resin composites for RLT, the choice of veneering material might influence the success of the iSC. From previous investigations of our research group, the authors observed that exemplarily Shofu Block HC (SH) and Grandio bloc (GB) present contrary chemical compositions and mechanical properties (Figure 1, Table 1).

This in-vitro study aimed to evaluate the aging and fracture resistance of iSCs fabricated with RLT by using two different CAD/CAM resin composites (SH, GB). For the frameworks, zirconia (ZrO_2_), PEEK, and cobalt–chromium (CoCr; control) were used. The null hypotheses were that there were no differences in aging performance and fracture force independent of the veneering material or the framework material.

## 2. Materials and Methods

To perform the test series, an abutment model was fabricated, on which the respective implant crown was attached. The model consisted of a laboratory implant (Medentika, Straumann, Basel, Switzerland; REF N51; LOT L0076219) and a milled, individually designed titanium abutment that was sandblasted (50 μm/2.0 bar). The abutment design was as follows: height 5 mm; diameter: 5.9 mm; angle 7.5 degrees; chamfer with anti-rotation. A molar crown (left first lower molar, n = 8 per group) was selected for the iSC. The implant crown was composed of a framework and a CAM-milled veneer structure [22]. The gaps between the abutment and the crown as well as between the framework and the veneering were set to 50 µm. Three framework materials and two CAD/CAM resin composites (A2 HT) were chosen as CAM-milled veneer structure materials. The layer thicknesses were: framework 0.6 mm; veneer circular 0.8 mm; occlusal 1.5 mm (Table 2).

Before the respective workpieces were adhesively luted, the adhesive surfaces were colored with a permanent marker (Edding 3000 black, Ahrensburg, Germany) to ensure that all surfaces would be properly blasted. The surfaces were sandblasted at an angle of 45° for 10 s at a distance of 10 mm: CoCr 250 μm/2.5 bar [23]; ZrO_2_ 50 μm/2.5 bar [24]; PEEK 110 μm/2.5 bar [8], and both CAD/CAM resin composites with 50 μm/2.0 bar [25,26]. After sandblasting, all workpieces were cleaned in an ultrasonic bath, dried with oil-free air, and a coupling agent was applied. For CoCr and ZrO_2_ frameworks, a coupling agent was applied for 60 s (Monobond Plus, Ivoclar Vivadent, Schaan, Liechtenstein; LOT Z0150N), and for PEEK frameworks, a primer recommended by the manufacturer (VisioLink, Bredent; LOT 204207) was used that was subjected to light polymerization for 90 s. For the CAM-milled veneer structure fabricated from Grandio blocs a coupling agent recommended by the manufacturer (Ceramic Bond, VOCO) was applied for 60 s. For veneer structures milled from Shofu blocks, a recommended bonding agent (HC Primer, Shofu; LOT 012009) was thinly applied and then light-cured for 20 s [26]. According to guidelines, a self-adhesive resin luting composite (RelyX Unicem 2 Automix, 3M, Saint Paul, MN, USA; LOT 7717107) was used to adhesively lute the veneer structure to the framework, which was light-cured for 20 s from each side [22,27]. The crowns were semi-permanently cemented to the abutment (Harvard Implant, Harvard, Hoppegarten, Germany; LOT 92103201), and the implants were embedded in Technovit 6091 (Kulzer, Hanau, Germany) in the same position by means of a previously fabricated appliance. After the specimens had been stored in deionized water (37 °C, 24 h), the crowns were loaded in a chewing simulator (TCML, 10,000 thermal cycles 5 °C to 55 °C for 120 s, 1.2 million, loading force 50 N) in a randomized order. After renewed storage in deionized water (37 °C, 24 h), the fracture force was determined by using a universal testing machine (Z010, Zwick, Ulm, Germany). To avoid force peaks, a 0.5 mm thick tin foil was placed between the specimen and the press plunger during each fracture load determination. A spherical, 6 mm diameter plunger applied the force centrally to the occlusal at a crosshead speed of 1 mm per minute. Failure was defined when the measured force of the load dropped 10% below the maximum point. 

Based on a previous study [8], the sample size was calculated assuming 95% power, which yielded eight restorations per group. Statistical analysis was performed by using descriptive statistics, Kolmogorov-Smirnov tests, one-way ANOVA, and Bonferroni post-hoc tests (SPSS 29.0, IBM, Armonk, NY, USA). The level of significance was set to α = 0.05.

## 3. Results

### 3.1. TCML

Five crowns did not survive the TCML (Table 3). Two crowns from ZrO_2_-SH and one from ZrO_2_-GB had no cement on the CAD/CAM resin composite surface, one crown from ZrO_2_-SH had less than 50% cement on the surface, and the surface of one CoCr-SH crown was largely covered with cement.

### 3.2. Fracture Force and Fracture Pattern

**Figure 2:**
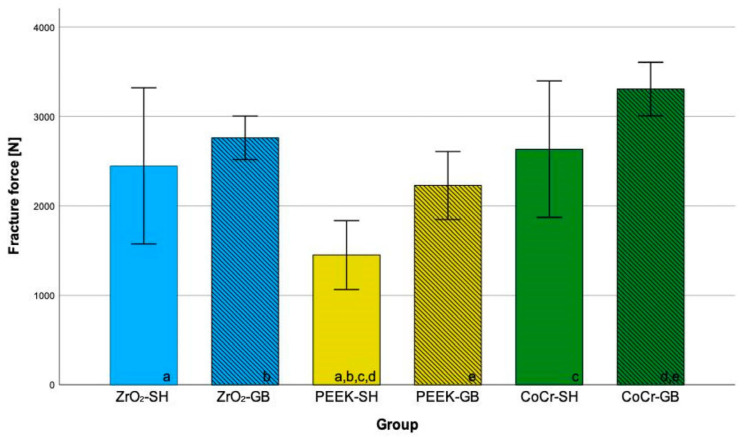
Fracture forces (mean, 95% confidence interval) of the tested materials for implant-supported crowns; frameworks: zirconia (ZrO_2_), polyetheretherketone (PEEK), cobalt–chromium (CoCr); CAM-milled veneer structure: Shofu Block HC (SH, plane), Grandio blocs (GB, lines); identical letters indicate statistically different results.

**Figure 3:**
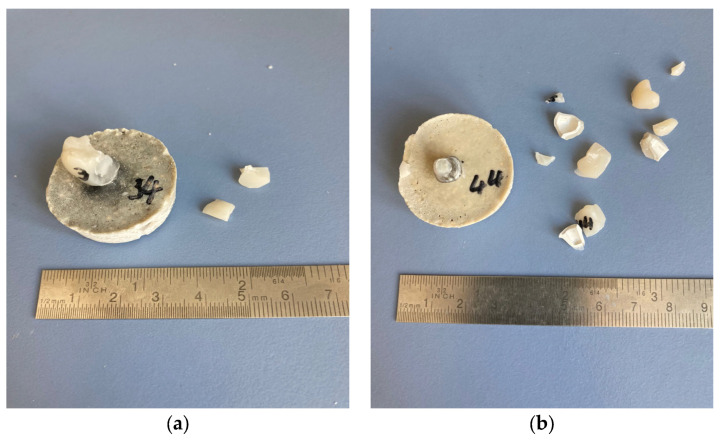
Example of fracture patterns of implant-supported single crowns with polyetheretherketone frameworks and (**a**) Shofu Block HC veneering (chipping) or (**b**) Grandio blocs veneering (framework fracture).

The measured forces were significantly different between the groups (normally distributed; ANOVA *p* < 0.001). The smallest fracture force was yielded in the PEEK group. The PEEK-SH group achieved significantly smaller values than both the CoCr and ZrO_2_ groups (*p* ≤ 0.031, Figure 2). When comparing similar framework materials, no significant differences for the veneering material were observed. Both CoCr frameworks and the ZrO_2_-SH never fractured, all PEEK-GB frameworks fractured (Figure 3). For ZrO_2_-GB and PEEK-SH events of chipping but also events of framework fractures occurred (Table 3).

## 4. Discussion

The veneering of iSCs with RLT might be a promising approach to avoid chipping in daily dental practice. The aim of this in-vitro study was to evaluate the aging and fracture resistance of iSCs fabricated with RLT by using two different CAD/CAM resin composites and three different framework materials. 

Four iSCs with zirconia framework and one with cobalt–chromium framework did not survive TCML due to decementation. The design of the abutment was chosen according to previous studies that described that an abutment height of 5 mm resulted in significantly higher pull-off forces than an abutment height of 3 mm [28,29]. Besides, it was observed that the application of the semi-permanent cement Harvard Implant combined favorable characteristics. It yielded relatively high pull-off forces in comparison to temporary cement and—compared to other semi-permanent cement or adhesive luting—enabled a non-destructive removal of high-strength ceramic restorations [28,29]. Nonetheless, decementation of the iSCs occurred in the present investigation, especially for zirconia frameworks. This might be explained by differences in surface roughness and geometric appearance of the intaglio surface of the framework materials that could have resulted from different manufacturing devices e.g., burs or milling machines.

Mean fracture forces were different between the framework materials, yet no significant differences between the two veneering materials with similar frameworks were observed. The fracture forces were highest for zirconia and cobalt–chromium frameworks and significantly lower for PEEK (BioHPP) frameworks, especially with SH veneer structures. Crowns with veneer structures from GB presented smaller standard deviations and higher fracture forces than those from SH. GB differs mainly from SH with respect to the filler content and the filler sphericity. The higher packing density of GB results in better mechanical properties [30,31]. Preis et al. investigated iSCs fabricated with RLT. They combined BioHPP frameworks with an ultra-low filled [32] CAD/CAM resin composite by Bredent (BreCAM.HIPC). They observed smaller mean fracture forces (1920 N) in comparison to PEEK-GB (2229 N) but higher values in comparison to PEEK-SH (1451 N) [33]. This might be explained by the differences in the filler contents with GB featuring relatively high filler content by mass. Ghodsi et al. also tested BreCAM.HIPC for iSCs fabricated with RLT in combination with CoCr, 3Y-TZP, and BioHPP as framework material. Zirconia yielded the highest fracture forces (2567 N). Surprisingly, iSCs from PEEK (2037 N) and CoCr (2032 N) frameworks presented no statistical difference [13]. For three-unit fixed dental prostheses, results are available for the combination of BioHPP frameworks and the ultra-low filled CAD/CAM resin composite by Bredent. They revealed similar 95% confidence intervals (1911–2124 N) in comparison to PEEK-GB but higher values in comparison to PEEK-SH [8].

Except for PEEK-GB, the fracture force of most iSCs was determined by the chipping of the veneer structure. Against this background, the choice of CAD/CAM resin composites for the veneer structure seems to impact the survival of iSCs during loading to failure. Thus, the choice of a material with a higher filler percentage by mass might be favorable for veneer structures. Nonetheless, the maximum loadings on molars in patients with bruxism were described with values of approximately 1000 N [34], which was yielded by every iSC. Therefore, limitations of iSCs fabricated from the aforementioned material combinations by using RLT should not be expected. 

The investigation is limited to the in-vitro character that did not include varieties of clinical loading situations. Moreover, in TCML steatite antagonists were chosen for better standardization instead of teeth. Besides, the used products and the standardized procedures for fabricating the crowns might be different from the clinical routine since, e.g., pretreatment procedures or application times recommended by the manufacturer might not be met in daily practice.

Within the limitations of the present investigation, the usage of implant-supported molar crowns with a CAD/CAM resin composite veneer structure might be a promising approach in dental practice and should be further investigated in in-vitro and in-vivo settings. The choice of CAD/CAM resin composite and of the framework material is attributed to the mechanical properties of the restoration.

## Figures and Tables

**Table 1:** Properties of the two CAD/CAM resin composites chosen for veneer structure.

Properties	Unit	Shofu Block HC	Grandio Blocs
Filler [16,17,18]			
Content	wt%	~62–63	~82–83
	Vol.%	~71–74	~52–53
Maximum filler size	µm	~11	~6
Sphericity	-	~0.71–0.83	~0.59–0.60
Chemical composition	-	Si, Zr	Si, Al, Ba
Phase composition	-	glass	glass
Monomer	-	UDMA, TEGDMA	Bis-GMA, UDMA, TEGDMA
Mechanical [19,20,21]			
Elastic modulus	GPa	8	17
Flexural strength	MPa	177–121	251
Vickers hardness	HV 0.2	87–88	149–152
Water uptake	µg/mm^3^	39.6	11.8

Computer-aided design/computer-aided manufacturing (CAD/CAM), urethane dimethacrylate (UDMA), triethylene glycol dimethacrylate (TEGDMA), bisphenol A-glycidyl methacrylate (Bis-GMA).

**Table 2:** Investigated materials: product name, code, LOT, and manufacturer.

	Code	Name	LOT	Manufacturer	Milling Device
Framework	CoCr	Ceramill Sintron	2012001	Amann Girrbach, Koblach, Austria	Ceramill Motion 2 (Amann Girrbach)
	ZrO_2_	Vita YZ HT	74420	Vita Zahnfabrik, Bad Säckingen, Germany	inLab MC X5 (Dentsply Sirona, Charlotte, SC, USA)
	PEEK	breCAM.BIOHPP	503576	Bredent, Senden, Germany	inLab MC X5 (Dentsply Sirona)
Veneer	GB	Grandio blocs	2117093	VOCO, Cuxhaven, Germany	inLab MC X5 (Dentsply Sirona)
	SH	Shofu Block HC	0121143	Shofu, Kyoto, Japan	inLab MC X5 (Dentsply Sirona)

**Table 3:** Survival, fracture force, and failure modes of the implant-supported crowns; frameworks: zirconia (ZrO_2_), polyetheretherketone (PEEK), cobalt–chromium (CoCr); CAM-milled veneer structure: Shofu Block HC (SH), Grandio blocs (GB).

Code	TCML Survival	Fracture Force in N				Number of Specimens with Failure Mode
		Mean (SD)	95% CI	Minimum	Maximum	
ZrO_2_-SH	5/8	2447 (702) ^a^	1574; 3319	1476	3196	5 chipping, 0 fracture
ZrO_2_-GB	7/8	2759 (264) ^b^	2515; 3003	2436	3090	4 chipping, 3 fracture
PEEK-SH	8/8	1451 (460) ^a,b,c,d^	1067; 1836	974	2081	6 chipping, 2 fracture
PEEK-GB	8/8	2229 (454) ^e^	1849; 2608	1333	2712	0 chipping, 8 fracture
CoCr-SH	7/8	2634 (825) ^c^	1871; 3397	1537	3526	7 chipping, 0 fracture
CoCr-GB	8/8	3305 (359) ^d,e^	3005; 3606	2961	4045	8 chipping, 0 fracture

Identical superscript letters indicate statistically different results in the column.

## Data Availability

Data is available from the authors upon reasonable request.
